# Diagnostic Challenges in Fungal Coinfections Associated With Global COVID-19

**DOI:** 10.1155/sci5/6840605

**Published:** 2025-05-07

**Authors:** Ariyo Shahin Jafari, Amir Sasan Mozaffari Nejad, Hossein Faraji, Ahmed S. Abdel-Moneim, Saeme Asgari, Hakime Karami, Ali Kamali, Ali Asghar Kheirkhah Vakilabad, Ali Habibi, Motahareh Faramarzpour

**Affiliations:** ^1^Department of Medical Parasitology and Virology, Sechenov University, Moscow, Russia; ^2^Bio Environmental Health Hazards Research Center, Jiroft University of Medical Sciences, Jiroft, Iran; ^3^Universal Scientific Education and Research Network (USERN) JMU Office, Jiroft University of Medical Sciences, Jiroft, Iran; ^4^Tropical and Communicable Diseases Research Center, Iranshahr University of Medical Sciences, Iranshahr, Iran; ^5^Department of Microbiology and Immunology, College of Medicine and Health Sciences, Sultan Qaboos University, Muscat, Oman; ^6^Department of Biochemistry and Biophysics, TeMS.C., Islamic Azad University, Tehran, Iran; ^7^School of Medicine, Jiroft University of Medical Sciences, Jiroft, Iran; ^8^Department of Accounting and Management, Islamic Azad University, Pardis Branch, Pardis, Iran

**Keywords:** COVID-19, fungal coinfections, intensive care units (ICUs), invasive fungal infections (IFIs), opportunistic infections, SARS-CoV-2

## Abstract

The early diagnosis of opportunistic infections is a critical concern for patient care worldwide, particularly in the context of the COVID-19 pandemic. This review examines the challenges and advancements in the management and early diagnosis of opportunistic fungal infections, which have become increasingly prominent during the pandemic. Using multiple sources, including curated databases such as PubMed and Scopus, as well as Google Scholar for broader literature searches, we systematically reviewed studies on COVID-19-associated fungal infections, with a focus on candidiasis, mucormycosis, and aspergillosis. The inclusion criteria encompassed peer-reviewed articles, clinical case reports, and cohort studies that discussed diagnostic methods, clinical outcomes, and treatment responses. Data were systematically extracted and analyzed to identify key trends and gaps in current diagnostic practices. Given the significance of opportunistic fungal infections—particularly the selected species—this review provides a comprehensive analysis of diagnostic challenges and advancements in the context of COVID-19 and beyond. Currently, there is no definitive strategy for effectively addressing these opportunistic pathogens, highlighting the need for continued research and innovation. Despite advancements in medical technology, opportunistic fungal infections continue to pose significant challenges to early and accurate diagnosis. The COVID-19 pandemic has exacerbated these challenges, with secondary fungal infections contributing to increased morbidity and mortality rates. This review highlights the complexities of diagnosing fungal coinfections and emphasizes the urgent need for improved diagnostic strategies. Enhancing the early and accurate detection of these infections is critical for effective patient management, particularly during viral pandemics. Addressing the challenges outlined in this review requires innovative diagnostic approaches to improve patient outcomes and reduce the burden of opportunistic infections on global healthcare systems.

**Summary**

• Diagnosing opportunistic fungal infections during the COVID-19 pandemic poses significant challenges.
• Accurate and timely fungal coinfection diagnosis is crucial to prevent increased mortality.
• The review the analyzed literature to understand challenges in fungal disease diagnosis.
• Innovative diagnostics are needed to improve patient care and reduce healthcare impact.

## 1. Introduction

Throughout history, the prevalence and recurring outbreaks of infectious diseases have had substantial and long-lasting consequences on society [1]. Coronaviruses (CoVs) constitute a diverse group of viruses that infect a wide range of animals and humans, causing illnesses that can range from mild to severe respiratory conditions. They are large, enveloped, positive-sense single-stranded RNA viruses (+ssRNA viruses) that infect various avian and mammalian species [2]. CoVs are characterized by the presence of spike-like projections of glycoproteins on their surface, which resemble a crown when viewed under an electron microscope; hence, they are termed coronaviruses.

Between 2002 and 2012, two highly pathogenic CoVs of common human–animal origin emerged: severe acute respiratory syndrome coronavirus (SARS-CoV) and Middle East respiratory syndrome coronavirus (MERS-CoV). These viruses caused respiratory diseases, including severe lower respiratory infections and pneumonia, posing significant health risks. Consequently, they transformed emerging CoVs into serious threats to public health [3–7]. A new CoV, SARS-CoV-2, emerged in Wuhan, China, in late 2019 and early 2020, leading to an outbreak of atypical viral pneumonia. This novel illness, referred to as coronavirus disease 2019 (COVID-19), spread rapidly worldwide due to its high transmissibility [5, 8, 9].

The current pandemic has far surpassed both SARS and MERS in terms of the number of cases and geographic extent, presenting a tremendous and devastating threat to global public health [5, 10, 11]. Despite the implementation of various treatment and control strategies, the emergence of new strains of COVID-19, including beta coronaviruses (β CoV), continues to be associated with morbidity and mortality rates. Secondary infections caused by a wide range of opportunistic bacterial and fungal pathogens, combined with an abnormal host immune response characterized by cytokine storms, contribute to increased mortality among patients infected with SARS-CoV-2 [12, 13].

## 2. Invasive Fungal Infections (IFIs) as a Public Health Concern

**Figure 1:**
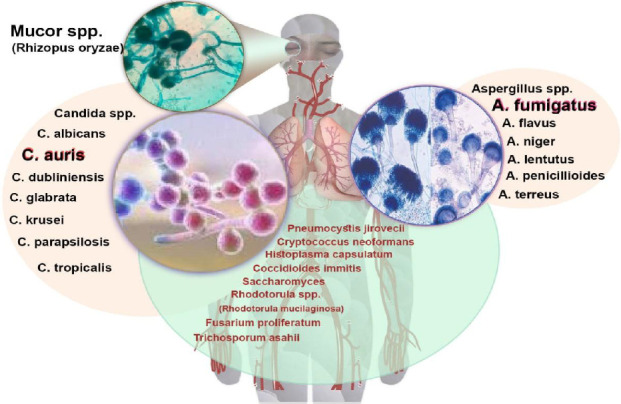
The principal fungal coinfections in patients with COVID-19.

IFIs have garnered significant attention as a public health concern in the past decade, owing to several high-profile outbreaks. Similar to other respiratory pathogens like influenza, fungal infections are emerging as a serious concern in patients, particularly during hospitalization, in intensive care units (ICUs), and even after recovery [14, 15]. In the ICU, mortality rates due to fungal infections have been reported to range from 5% to 70% [14]. The IFI incidence rate has been reported to be between 4% and 27.7% for COVID-19 [16]. Although several fungal species have been identified in hospitalized COVID-19 patients, *Candida*, *Mucor*, and *Aspergillus* species are the primary fungal microbes responsible for coinfections in patients with severe COVID-19 caused by SARS-CoV-2 [17, 18] (Figure 1).

In patients with pulmonary infections caused by *Aspergillus*, rhino-orbital-brain involvement is induced by *Mucor*, while upper gastrointestinal infections are associated with *Candida*, and bloodstream infections are linked to *Candida* dissemination (Figure 1) [19]. In addition to mucormycosis, meningoencephalitis caused by *Cryptococcus neoformans* has also been reported [20]. The diagnosis of histoplasmosis should be considered in endemic countries, particularly among individuals with significant tourist mobility to and from these regions; some sources even suggest its increasing prevalence [21, 22].

*Pneumocystis jirovecii,* although rare and infrequently reported, has been observed in pulmonary infections associated with SARS-CoV-2 [23, 24]. Infection with *Saccharomyces cerevisiae* has been documented in ICU patients with COVID-19 following *Saccharomyces* supplementation [25]. *Fusarium* may pose complex challenges even for patients with CoV whose immune systems are not severely compromised [26]. *Rhodotorula mucilaginosa* can be present either alone or in conjunction with species such as *Candida glabrata* [27]. Coinfection with *Trichosporon asahii* and COVID-19 has been reported in the form of nosocomial pneumonia or fungemia [27, 28].

## 3. Mucormycosis

Mucormycosis, also known as zygomycosis or “black fungus,” is a serious angioinvasive fungal infection. It is caused by a group of molds known as Mucoromycetes, which belong to the subphylum *Mucoromycotina* and the order *Mucorales*. The most common fungi responsible for mucormycosis include *Rhizopus* spp., *Mucor* spp., *Rhizomucor* spp., *Syncephalastrum* spp., *Cunninghamella bertholletiae*, *Apophysomyces* spp., and *Lichtheimia* (formerly *Absidia*) spp [29]. Notably, *Rhizopus oryzae* accounts for approximately 60% of cases and 90% of the rhino-orbital-cerebral mucormycosis (ROCM) variant [30].

**Figure 2:**
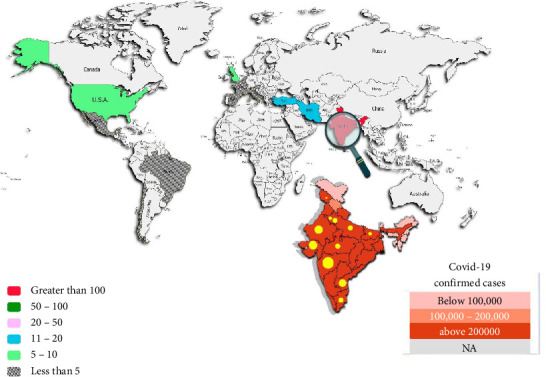
Global distribution of COVID-19-associated mucormycosis.

Mucormycosis is associated with several underlying conditions that increase susceptibility to infection, including prolonged neutropenia, hematologic malignancies, uncontrolled diabetes, iron chelation therapy, and skin breaches. These factors significantly elevate the risk of invasive disease, particularly in non-COVID-19 patients [31]. The emergence of mucormycosis has complicated the management of COVID-19, especially among critically ill patients on mechanical ventilation, who are at heightened risk for fungal infections. In March 2021, 41 cases of COVID-19-associated mucormycosis (CAM) were reported globally, with India accounting for 70% of these cases. The second wave in India saw a surge, with Maharashtra reporting 2245 cases and 120 fatalities, and Rajasthan documenting 2651 cases by June 2021 [32]. A study of 102 CAM cases indicated that 69.6% of patients were male, and 70.5% had concurrent COVID-19. Steroid use was prevalent in 68.6% of cases, while diabetes mellitus affected 88.2% of patients. Mucormycosis primarily impacted the sino-nasal area (72.5%), with a recorded mortality rate of 23.5% [33]. The incidence of CAM is particularly high among diabetic patients, with approximately 95% of severe COVID-19 cases affected [32]. The predominant causative agent in India is *Rhizopus arrhizus*, along with other species such as *Mucor irregularis* and *Apophysomyces variabilis* [34–36]. The number of countries reporting CAM has been discussed in a previous study (Figure 2) [37].

In India, the prevalence of mucormycosis is significantly higher than in developed countries, exacerbated by the widespread use of immunosuppressants and corticosteroids for COVID-19 treatment [29, 38]. Additionally, both hyperglycemia and COVID-19 can disrupt iron metabolism, further increasing the risk of mucormycosis [39]. Diagnosing and treating mucormycosis in COVID-19 patients poses significant challenges, necessitating faster diagnostic methods and alternative treatment options [40, 41]. Excessive reliance on conventional precautions may disrupt nasal microbiomes, thereby increasing the risk of fungal infections [42]. Interestingly, increased zinc consumption, aimed at preventing viral infections, may inadvertently inhibit fungal growth, suggesting a potential therapeutic avenue [43]. The diversity of fungal species involved, such as *Rhizopus microsporus* and *Lichtheimia corymbifera*, highlights the complexities of managing mucormycosis [44]. Recognizing these factors enables clinicians to effectively address the challenges posed by mucormycosis during the COVID-19 pandemic.

## 4. Aspergillosis

Aspergillosis encompasses numerous mold species that are prevalent in diverse climates globally, frequently isolated from both outdoor and indoor environments, including tertiary care centers. It is a leading cause of life-threatening fungal infections. Despite significant advancements in diagnosing and treating aspergillosis, effectively preventing and managing severe hospital-acquired fungal diseases remains challenging, with high mortality rates, especially among immunocompromised patients affected by invasive infections [45–47].

Infections are typically caused by *Aspergillus fumigatus* (roughly 90%), followed by *A. flavus*, *A. nidulans*, *A. niger*, *A. terreus*, and *A. versicolor* [48]. The prevalence of aspergillosis has increased due to modern medical practices and a rising number of individuals with compromised immune systems from cancer treatments, organ transplants, and prolonged immunosuppressive therapy. It manifests in several forms: invasive pulmonary aspergillosis (IPA), chronic pulmonary aspergillosis (CPA), allergic bronchopulmonary aspergillosis (ABPA), chronic rhinosinusitis (CRC), fungal asthma, and *Aspergillus* bronchitis [49]. Notably, IPA incidence has risen 10-fold in the past two decades [50].

IPA is a recognized complication in severely immunosuppressed individuals, such as those undergoing hematopoietic transplantation or chemotherapy, and is associated with high mortality rates [49]. Studies indicate that patients with severe acute respiratory distress syndrome (ARDS) from influenza are also at risk for rapid progression to IPA, linked to prolonged hospital stays and higher mortality [51]. Other risk factors include liver cirrhosis, systemic connective tissue diseases, chronic kidney disease (CKD) or renal replacement therapy (RRT), influenza-associated aspergillosis (IAA) [51, 52], advanced solid cancers, and diabetes mellitus [53].

Recent diagnostic advancements show that approximately 50% of IPA cases occur in ICUs, often in patients not experiencing neutropenia [49, 51]. The association between ARDS and IPA suggests that alveolar damage may facilitate fungal invasion [54]. COVID-19-associated pulmonary aspergillosis (CAPA) is a significant concern, with *A. fumigatus* identified as the primary coinfecting species in patients with COVID-19, alongside *A. flavus* and *A. terreus* [55].

Predisposing factors for CAPA include diabetes, immunomodulatory therapies, prior respiratory diseases, cardiovascular disease, hepatic disease, renal failure, prior use of antibiotics and antifungal treatments, as well as environmental and logistical factors. Similar to other forms of pulmonary aspergillosis, CAPA requires underlying host factors such as allergic conditions (e.g., asthma), airway diseases (e.g., bronchial dilation, cystic fibrosis), chronic lung cavities (e.g., tuberculosis, sarcoidosis), or immune deficiency [53].

## 5. Candidiasis

*Candida* is a genus of yeast comprising approximately 200 species, including *Candida albicans*, *C. glabrata*, *C. parapsilosis*, *C. tropicalis*, and *C. krusei*, commonly found on mucosal surfaces [56, 57]. While *Candida* can infect both immunocompetent and immunocompromised individuals, infections are more prevalent in those with weakened immune systems, leading to candidiasis being referred to as the “disease of the diseased” [58]. *Candida* species can cause a range of clinical symptoms, from mucocutaneous overgrowth to severe disseminated infections such as candidemia. Although *Candida albicans* is frequently found in humans, it does not always cause symptoms. However, it can lead to various chronic and acute opportunistic infections in immunocompromised individuals, including diabetics, organ transplant recipients, and people living with human immunodeficiency virus (HIV), manifesting as thrush, vaginitis, cutaneous infections, and nail infections [59]. Notably, the incidence of infections caused by non-*albicans Candida* (NAC) species is increasing, with some exhibiting resistance to standard antifungal treatments. Several studies have reported on *C. albicans*, *C. parapsilosis*, *C. tropicalis*, *C. glabrata*, *C. guilliermondii*, and *C. krusei*, noting their occasional resistance to conventional antifungal treatments [59, 60]. One study at Siriraj Hospital in Thailand analyzed data from 156 candidemia patients between January 2016 and December 2017, finding that 71.2% had candidemia caused by NAC species, reflecting an increasing prevalence in the region [61]. Additionally, research indicated that *Pichia kudriavzevii*, *C. dubliniensis*, and *C. glabrata* exhibited decreased susceptibility to azoles [62].

Various virulence mechanisms enable *Candida* species to transition from harmless commensals to pathogens, including adherence to host tissues, biofilm formation, and the production of extracellular hydrolytic enzymes [63]. Factors contributing to this transition include severe immunosuppression, prematurity, broad-spectrum antibiotic use, empirical antifungal therapy, diabetes, renal failure, and the presence of catheters, as well as major surgery, and burns [64]. While clinical symptoms of infections caused by NAC spp. are often similar, some NAC spp. exhibit inherent resistance to antifungal medications or can develop resistance [65]. *Candida* spp., especially from NAC species, is a common ICU pathogen, infecting 6% to 10% of patients. The incidence of candidemia is increasing, with invasive candidiasis responsible for 19% to 40% of all deaths and a mortality rate exceeding 70% among ICU patients [66]. *Candida auris* has emerged as a significant NAC pathogen since 2009. It belongs to the Clavispora clade of the Metschnikowiaceae family [67, 68]. The phylogenetic relationship of *C. auris* with other *Candida* species remains unclear due to the rarity of some related species; however, five clades have been identified, linked to species such as *C. haemulonii*, *C. pseudohaemulonii*, *C. duobushaemulonii*, and more recently, *C. heveicola* [69, 70].

*C. auris* infections have been reported in over 40 countries, affecting 5% to 10% of infected individuals, ranging from mild infections like otitis media to severe invasive candidiasis. Its epidemiology is similar to that of other *Candida* species [71]. Initially, *Aspergillus fumigatus* was seen as the main cause of fungal superinfections in critically ill COVID-19 patients, but candidiasis has become increasingly significant. This shift is due to factors like antibiotic and corticosteroid use, central venous catheters, and SARS-CoV-2 damage in ARDS patients. *Candida albicans* is the most common yeast in invasive infections among these patients, followed by *C. auris*, *C. glabrata*, *C. parapsilosis*, *C. tropicalis*, *S. cerevisiae*, and *Rhodotorula* spp [72]. In New Delhi, *C. auris* was responsible for 67% of candidiasis cases [73]. It was first reported in Salvador, Brazil, in December 2020 [74], with subsequent cases in Mexico [75]. The role of *C. auris* in patients and its transmission via respiratory equipment has been highlighted in several studies [76]. Other species, including *C. glabrata*, *C. dubliniensis*, *C. parapsilosis*, *C. tropicalis*, and *C. krusei*, have also been identified. In COVID-19 cases, various *Candida* species have been isolated from candidemia [77, 78].

Early and accurate identification is crucial for effective treatment of opportunistic fungal infections, but diagnostic challenges complicate detection for healthcare professionals.

## 6. Challenges in COVID-19-Associated Fungal Disease

Diagnosing fungal infections based solely on clinical signs, symptoms, and nonspecific radiographic manifestations can be challenging. Laboratory diagnosis of fungal diseases typically involves microscopic examination, culture, antigen or antibody testing, and molecular assays [79–81]. Direct microscopic examination requires sampling from the site of infection; however, identifying the genus or species based solely on microscopic features may not always be feasible or sufficient. Additionally, culture techniques can be insensitive and time-consuming. Fungi are also part of the human microbiome, which limits the effectiveness of antigen- and antibody-based assays, as fungal colonization can lead to false-positive results [82].

Moreover, routine serologic tests for emerging fungi are often unavailable, and current diagnostic methods require specialized mycological expertise. As the diversity of fungi that clinical mycologists must identify increases, there is a pressing need for innovative identification approaches beyond traditional phenotypic methods. Consequently, molecular techniques, particularly polymerase chain reaction (PCR) and antigen detection, are gaining prominence as alternatives to culture-based diagnostics for fungal infections [81, 83–85]. PCR, which targets the highly variable internal transcribed spacer (ITS) regions of fungal ribosomal DNA (rDNA), is a valuable tool for distinguishing fungal species [86]. However, challenges persist, especially in diagnosing IFIs, which can remain undetected until postmortem in some cases [87].

This review will address the diagnostic challenges associated with opportunistic fungal infections frequently observed during the COVID-19 pandemic.

## 7. Diagnosis of CAM

Diagnosis of CAM, due to its rapid invasion and spread throughout the body, affecting most vital organs, should begin at the time of hospitalization [88, 89]. This process should take into account the patient's history, including underlying conditions and other risk factors. Mucormycosis can be categorized based on its location: pulmonary, gastrointestinal, cutaneous, disseminated, and especially ROCM [29]. ROCM typically manifests as sinusitis in the ethmoidal or sphenoidal sinuses and can progress to cavernous sinus syndrome or thrombosis of the internal carotid artery [90].

Necrotic ulcers resulting from osteomyelitis of the facial bones can impair the optic and cranial nerves, leading to headaches and facial discomfort. Diagnostic samples, including scrapings from the upper turbinates, aspirated sinus material, sputum, and biopsy specimens, can be examined using 10% potassium hydroxide (KOH) or optical brighteners like Blankophor and Calcofluor [85, 91, 92]. Mucorales are characterized by thick-walled, irregular, aseptate, and refractile hyphae measuring 6 to 25 μm in diameter, often exhibiting swollen and twisted forms with common 90° bifurcations [93, 94]. Histological analysis may reveal acute suppurative and localized granulomatous inflammation. Fungal elements are readily visible on hematoxylin and eosin-stained sections, while periodic acid-Schiff or Grocott's methenamine silver staining enhances the visibility of hyphae, particularly in cases of vascular invasion leading to thrombosis and infarction [85, 92].

Direct microscopic analysis and histopathological examination for Mucorales detection can present diagnostic challenges, as these fungi may be difficult to differentiate from *Aspergillus* hyphae. Mucorales grow rapidly (3 to 7 days) on standard fungal culture media (e.g., potato dextrose agar and Sabouraud agar) at 25°C to 30°C; however, only about 50% of cases may yield positive cultures despite positive microscopy findings [95, 96]. Coinfection of the lungs by *Rhizomucor* spp., and SARS-CoV-2 can occur, resulting in symptoms like dyspnea, cough, and airway bleeding [37].

Pulmonary mucormycosis (PM) primarily affects the bronchial airways and lung parenchyma, with potential extension into the chest wall, leading to complications such as cavitation and pericarditis [97]. An early diagnostic feature of PM is the reverse-halo sign on computed tomography (CT) scans, although this finding is rare in COVID-19 cases. Magnetic resonance imaging (MRI) effectively captures soft tissue and evaluates disease severity [98–100].

Some cases may show subtle thickening of the sinus mucosa or extraocular muscles without visible abnormalities in the sinus bones, despite clinical symptoms. A hypointense rim around lesions may indicate the accumulation of blood products or metals like iron and magnesium due to fungal activity [101].

Rhino-cerebral mucormycosis often progresses with orbital invasion, increasing the risk of cavernous sinus infections and internal carotid thrombosis, which can elevate mortality rates. A “black hole” sign, representing a lack of contrast uptake by lesions, has been noted [102]. In patients with COVID-19, whether alone or in conjunction with mucormycosis, abnormal levels of nonspecific laboratory markers associated with COVID-19-related sepsis have been observed, including elevated lactate dehydrogenase, C-reactive protein, D-dimer, and renal profile derangements, along with cytopenia [98, 103–105].

For bacterial and yeast infections, matrix-assisted laser desorption/ionization time-of-flight mass spectrometry (MALDI-TOF MS) is a rapid and reliable method for detecting culture isolates. However, the handling of filamentous fungi presents greater challenges, necessitating several pre-analytical procedures. Despite these challenges, researchers have successfully employed MALDI-TOF MS technologies for the identification of Mucorales isolates [106–109].

Currently, molecular detection of Mucorales primarily relies on DNA detection, as β-D-glucan and galactomannan (GM) assays are insufficient for identifying this fungus [80, 110]. Initially, the detection of Mucorales DNA (specifically 18S rRNA) from clinical specimens served as a complementary tool to histopathological and microbiological diagnoses [111, 112]. Assays have been developed to detect Mucorales DNA in blood and urine samples [113, 114]. Notably, the levels of circulating Mucorales DNA in serum are significantly elevated compared to those in invasive aspergillosis (IA), likely due to the angioinvasive nature of Mucorales infections [113, 115].

The quantity and extractability of fungal DNA in clinical samples, influenced by sample type, greatly impact the sensitivity of DNA-based diagnostic methods. Formalin-fixed paraffin-embedded tissue (FFPET) exhibits lower DNA extractability compared to fresh tissue due to the detrimental effects of formalin on DNA [116]. This partial fragmentation of DNA reduces the sensitivity of PCR detection [80, 117]. Most DNA-based detection technologies utilize PCR to amplify target genetic material, resulting in high analytical sensitivity, which also increases the risk of infection by common environmental fungi [79, 80, 118]. Researchers and medical professionals have employed various diagnostic methods, including quantitative PCR, reverse transcription PCR (RT-PCR), and real-time PCR, to evaluate the performance of CAM diagnostics. These approaches have yielded both successful outcomes and occasional limitations or failures [113, 119].

## 8. Diagnosis of CAPA

**Figure 3:**
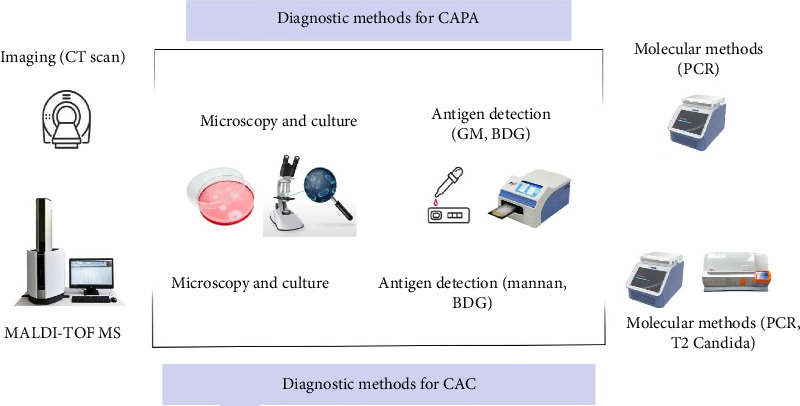
Diagnostic methods for COVID-19-associated fungal infections: CAPA and CAC.

The diagnosis of CAPA relies on the patient's medical history and clinical findings. Symptoms of IA are often nonspecific, with a lack of response to empiric antibiotic treatment being a common indicator. C-reactive protein (CRP) and procalcitonin (PCT) are nonspecific inflammatory markers useful for assessing treatment efficacy post-diagnosis [120, 121]. Diagnostic methods for IA include imaging, microscopy, histopathology, culture, antigen detection, and DNA detection [122] (Figure 3).

Microscopic examination is a straightforward method for detecting IA, but *Aspergillus* species rarely sporulate in vivo, leading to potential misidentification. Sensitivity varies widely (0%–90%) due to differences in techniques and mycologist expertise [123–126]. A positive culture from the same specimen is required for confirmation, as visible hyphae alone are insufficient. Obtaining biopsy samples for pulmonary IA can be risky due to low platelet counts. *Aspergillus* hyphae are thin (1–3 μm) and septate, complicating differentiation from other fungi such as *Scedosporium* and *Fusarium* species [122].

Historically, culture was the standard diagnostic method before the introduction of GM and PCR assays [127]. *Aspergillus* typically grows rapidly (within 48 h) on various media, but culture sensitivity is limited regarding infection versus colonization [122]. Characteristic CT findings for IA include reticular opacities and halo signs, but these are not specific and can overlap with other infections (such as *Scedosporium* spp. or *Cryptococcus* spp.), including COVID-19-associated PM [128].

Immunosuppressed patients receiving glucocorticoids may present with *Aspergillus* bronchopneumonia and varied CT abnormalities, including multiple nodules, lobar infiltrates, and widespread ground-glass opacities. The radiographic signs of these peribronchial zones of consolidation are non-specific and can resemble findings from other infections [129]. CT, particularly when combined with high-resolution CT (HRCT), alongside other diagnostic modalities, is valuable for diagnosing IPA. However, CT imaging can obscure parenchymal abnormalities indicative of CAPA. Detecting IA in mechanically ventilated COVID-19 patients can be challenging due to nodular infiltrates that complicate the identification of surrounding halos [130]. This challenge is exacerbated in severe COVID-19 pneumonia, where CT typically reveals bilateral peripheral ground-glass opacities, consolidation, and bronchovascular thickening. Consequently, distinguishing IA radiological signs from those associated with severe COVID-19 pneumonia is significant [131]. Patti et al. described a mechanically ventilated COVID-19 patient with newly developed thin-walled cavitary lesions populated by fungal ball-like lesions on CT [131]. Additionally, the transport of critically ill patients poses risks, making CT scans not always feasible [132].

GM assays, similar to those for diagnosing mucormycosis, are also unreliable. Other fungi that produce GM include *Penicillium*, *Fusarium*, *Alternaria*, *Histoplasma*, and *Cryptococcus* [122]. Swanink et al. noted that *Candida* spp. can yield low GM assay responses, possibly due to hydroxychloroquine (HCQ) use in COVID-19 treatment protocols [133]. The percentage of neutropenic individuals with IPA who test positive by culture ranges from 10% to 58% [122, 134]. *Aspergillus* species exhibit a high glucan content in their cell walls, particularly β-D-1,3-glucan (BDG) [135]. A study by Digby et al. found elevated BDG levels in ICU patients with bacterial and fungal infections compared to those without infections [136].

Some studies suggest that early BDG detection during infection progression is more likely than GM detection [137]. The BDG assay has traditionally been used in hematology patients at risk for IPA due to neutrophil deficiency, but it has also shown utility in critical care [138] and COPD patients [139]. In an animal model of IA, the BDG assay demonstrated earlier positivity in bronchoalveolar lavage (BAL) samples compared to serum, with greater overall sensitivity [140]. However, serum BDG also plays a role. Due to limited literature, uncertainty remains regarding its utility in diagnosing CAPA [141]. Despite these challenges, GM measurement techniques are still regarded as the gold standard in many laboratories, including at the PCR level.

## 9. COVID-19-Associated Candidiasis (CAC) Diagnosis

Physical symptoms, microscopic inspection, and blood cultures are the most common methods for diagnosing candidiasis and candidemia. Recently, multiple instances of oral candidiasis have been documented in COVID-19 patients, raising concerns about increased morbidity and mortality. Early detection of oral candidiasis in these individuals is critical for effective treatment [142]. However, relying solely on clinical criteria is insufficient, as many symptoms are not pathognomonic and are often identified only when treatment becomes difficult. A negative result does not exclude the possibility of infection. Microscopic inspection is a rapid and beneficial diagnostic tool [81, 143]. Blood cultures are considered the gold standard for diagnosing candidiasis, particularly in severe cases of candidemia or invasive candidiasis [144] (Figure 3).

However, fungal cell counts in infected tissues or circulating in the bloodstream are usually low, which can hinder the detection of *Candida* spp. using simple blood culture tests. As a result, more invasive procedures may be required [17, 27, 72]. Notably, only 50%–70% of patients with *Candida* bloodstream infections yield positive blood cultures [145]. Additionally, blood cultures may be negative in some COVID-19 patients, especially if drawn after the onset of *C. auris* fungemia [76]. Traditional phenotypic techniques have led to misidentification of *C. auris*, commonly mistaking it for *C. sake*, *C. duobushaemulonii*, *Rhodotorula glutinis*, *C. haemulonii*, or other *Candida* species [146]. *C. auris* can thrive in specific media, such as Sabouraud broth, at elevated temperatures (40°C–42°C) or in high salt concentrations (10%) [147–149].

On CHROMagar *Candida* medium, *C. auris* typically forms pink colonies, making it challenging to differentiate from *C. glabrata* and various other yeast species, including those in the *C. haemulonii* complex [150, 151]. Importantly, *C. auris* can switch between different colony phenotypes, such as pink, white, and dark purple [147, 151]. A novel chromogenic selective medium, CHROMagar™ *Candida* Plus, has been developed that allows for the differentiation of *C. auris*, which forms distinct cream-colored colonies with a blue halo after 48 h of incubation at 37°C [152]. CHROMagar supplemented with Pal's medium has also proven useful for distinguishing *C. auris* from *C. haemulonii* [153]. Automated yeast identification systems commonly misidentify *C. auris* isolates as *C. haemulonii* or *Rhodotorula glutinis* [47, 151]. An FDA-approved automated molecular test, the T2Candida nano-diagnostic panel, utilizes magnetic resonance technology to directly detect *Candida* spp. in whole blood samples within 5 h. This test targets the five most prevalent pathogenic *Candida* spp., including *C. krusei*, *C. glabrata*, *C. tropicalis*, *C. albicans*, and *C. parapsilosis*, and can also detect *C. auris* [154, 155].

Confirmation of *C. auris* infection requires additional methods. MALDI-TOF MS technology can rapidly identify yeast species directly from blood cultures within minutes, eliminating the need for sub-culturing [154]. Other techniques, such as BDG assays and mannan antigen testing, are useful for detecting deep-seated infections [154, 156]. Commercial enzyme-linked immunosorbent assay (ELISA) kits can detect *Candida* mannan antigen (MN) in serum samples, offering relatively high specificity and sensitivity [157]. It is important to note that BDG assays are not species-specific and may yield false positives. Although PCR assays on blood samples are not FDA-approved for diagnostic use, they can identify specific *Candida* spp. [17, 158]. In patients suspected of having invasive candidiasis, elevated levels of MN in the blood have demonstrated a sensitivity of 58% and a specificity of 93% [157, 159]. Blood PCR has shown pooled sensitivity and specificity of 95% and 92%, respectively, for detecting proven or probable invasive candidiasis compared to at-risk controls [72, 160].

The T2Candida Panel, which amplifies the ITS2 region, directly detects *Candida* spp. in EDTA blood samples within 5 h and has demonstrated efficacy in diagnosing candidemia and intra-abdominal candidiasis. However, its technical requirements may pose challenges [155, 158, 161]. To enhance sensitivity, combining multiple diagnostic techniques is recommended [27, 154, 156, 159]. The ITS-PCR assay allows differentiation of *C. auris* from *C. haemulonii* based on amplicon size [162]. Another approach utilizes direct PCR targeting glycosylphosphatidylinositol (GPI) protein–encoding genes to speciate *C. auris* and 18 other *Candida* species by visualizing different-sized amplicons on a gel [163]. Evaluation of a proficiency panel of 44 *C. auris* and 97 other yeast isolates achieved 100% accuracy (100% sensitivity and specificity) [164]. This assay has been modified to enable direct detection of *C. auris* from patient swabs, demonstrating 93% sensitivity and 96% specificity [165]. Several laboratories have developed real-time PCR tests for *Candida auris*, with ongoing research in this area [166–169].

The challenges of fungal infections in the context of COVID-19 extend beyond previous concerns. Patients may experience multiple concurrent fungal infections, complicating the differentiation from bacterial infections and viral superinfections. This multiplicity can hinder the diagnosis of other abnormalities. For instance, coinfections like mucormycosis and oral candidiasis require skilled mycologists to accurately identify based on clinical signs, medical history, and lab findings. Proper sample collection is crucial; some require microscopic examination, while others are cultured, with additional assays like spectrometry and molecular testing. However, challenges arise, particularly with mucormycosis and candidiasis coinfection, where biopsy is necessary due to the delicate nature of mucoromycetes and the risk of spreading infection. Furthermore, corticosteroid treatment can render tissues extremely thin and fragile, complicating the diagnosis and management of these infections [142].

In another scenario, a diabetic man with COVID-19 in the ICU was diagnosed with concurrent pulmonary aspergillosis (*Aspergillus fumigatus*) and mucormycosis. He underwent comprehensive diagnostics and received multiple antifungal and bacterial therapies. On the ninth day, he developed methicillin-resistant *Staphylococcus aureus* (MRSA) and *Klebsiella pneumoniae* ventilator-associated pneumonia. By day 36, despite requiring ventilator support, he tolerated intravenous liposomal amphotericin B (L-AmB) therapy and was eventually transferred to a long-term acute care facility [39]. Another case involved a 78-year-old male with type 2 diabetes mellitus and ketoacidosis, who presented with facial swelling, paralysis, ptosis, and a necrotic palatal ulcer. No COVID-19 coinfection was noted. CT imaging revealed a right maxillary sinus abscess, and cultured secretions identified *A. fumigatus*. Pathological examination confirmed mucormycosis. The patient received voriconazole, amphotericin B deoxycholate, and surgical debridement but unfortunately did not survive. In immunocompromised individuals, the coexistence of rhino-cerebral mucormycosis and aspergillosis should be considered for early treatment and improved prognosis [170]. A 69-year-old woman with a history of hypertension and diabetes presented with cerebro-rhino-orbital mucormycosis and an aspergillosis coinfection. The challenges of early detection of invasive mucormycosis highlight the importance of considering concomitant infections like aspergillosis. Fortunately, the patient was discharged after a successful recovery [171].

## 10. Economic Impact of Pandemics on Global Healthcare Systems

Given the aggressive nature of fungal pathogens, particularly mucormycosis, and their resistance to antifungal therapies, timely diagnosis is crucial for effective management and related research. While we did not find a specific article addressing the importance of timely interventions in *Aspergillus* and *Candida* infections, healthcare professionals must remain vigilant about potential false negatives and employ various diagnostic techniques, including molecular approaches, to achieve more accurate results.

These factors necessitate the availability of competent healthcare personnel and well-equipped laboratory facilities, which can be particularly challenging in low- and middle-income countries (LMICs). Notably, the ongoing COVID-19 pandemic has significantly affected LMICs, especially in regions such as Asia, sub-Saharan Africa, and South America. Despite efforts to enhance healthcare in these areas, a substantial portion of the population still lacks access to adequate diagnostic and treatment services, as well as sufficient medical personnel. The economies of nations worldwide have been considerably impacted by the COVID-19 pandemic, resulting in both short-term budgetary effects and long-term economic consequences. Measures such as quarantine, health facility preparedness, isolation of infected individuals, and contact tracing require significant public health resource allocation, human resources, and implementation costs. Additionally, healthcare expenses associated with antibiotics, medical supplies, personal protective equipment, and consumables further strain resources. Pandemics can lead to fiscal stress by reducing tax revenues while increasing expenditures, particularly in LMICs, where fiscal constraints are more pronounced and tax systems require further development. For instance, during the Ebola virus outbreak, the loss of government income due to quarantine and curfews severely impacted Liberia's economy, leading to increased public health spending, economic downturns, and reduced revenue. Economic shocks are common during pandemics, exacerbated by labor shortages across various professions, including diagnostic and treatment specialists, alongside increased mortality among both the general population and healthcare workers [172, 173]. Additional challenges include transportation disruptions, workplace closures, trade and travel restrictions, and border closures.

Together, these factors lead to decreased access to specialized care and a sharp rise in the cost of diagnostic kits and research budgets aimed at combating the CoVs. In such circumstances, the risk of other infections, including opportunistic fungal infections, which can lead to significant outbreaks or complicated treatment cases, is often overlooked. These challenges may be further exacerbated in tropical and subtropical regions (e.g., CAM in India). Thus, alternative methods like MALDI-TOF MS, which has a direct cost of only $1403.20 or $35.08 per isolate, resulting in a 74.0% cost savings, can benefit not only LMICs but also numerous clinics and laboratories in more affluent countries facing budget constraints [174]. More expensive methods, such as PCR, may not be reliably feasible. It is important to note that these fungi are typically saprophytic and present in the environment. For instance, *Aspergillus* spores, which are ubiquitous even in controlled environments, can contaminate molecular detection processes and yield false-positive results, raising questions about the accuracy of these molecular methods.

*Candida auris* further complicates this issue. Since its initial identification in 2009 from the ear discharge of a 70-year-old Japanese patient, *C. auris* has spread rapidly worldwide [69]. This species exhibits unique characteristics, including the ability to form biofilms on various surfaces, facilitating nosocomial transmission. *C. auris* has been isolated from catheters, central and peripheral line tips, and neurological shunts, among other indwelling medical devices [151]. Moreover, *C. auris* can persist in hospital environments for months by forming “dry” biofilms on environmental surfaces and equipment, such as reusable temperature sensors. Its biofilm-forming capability contributes to its role as a persistent colonizer and a challenging pathogen to eradicate within healthcare facilities [151]. Transcriptional studies have shown that biofilm development confers protection against antifungal medications and detergents, with mature biofilms (aged 24 h) demonstrating resistance to triazoles, polyenes, and echinocandins [175, 176].

As a result, the morphological features and initial diagnosis of this fungus can present challenges for laboratory personnel. Concerning CAM, it is noteworthy that many patients receiving medical treatment, but not on oxygen therapy, have been infected and diagnosed with mucormycosis. However, there does not appear to be a definitive link between oxygen therapy and susceptibility to this infection [177]. In general, it is crucial to adhere to established protocols, maintain distance, and limit the use of supportive devices, such as mechanical ventilator. While these measures are ideal in theory, the reality of managing severe COVID-19 cases, a surge of patients requiring urgent hospitalization, and overwhelmed medical staff under significant stress makes it challenging to observe all principles for addressing fungal species that are likely to be resistant to fungicides or that may rapidly develop resistance. Timely detection and differentiation of opportunistic fungal species from other infectious agents pose significant challenges. Currently, no studies have examined the effects of corticosteroids, immunomodulators, and other risk factors on the morphology of hyphae, spores, conidia, and yeasts. However, based on the authors' experience with dermatophytic fungi, this may also present moderate challenges.

## 11. Conclusion

IFIs have become a significant public health issue, particularly highlighted by recent outbreaks. These infections pose serious risks to hospitalized patients, especially in ICUs, with mortality rates ranging from 5% to 70% [14]. During the COVID-19 pandemic, the incidence of IFIs among patients with severe SARS-CoV-2 infection was reported between 4% and 27.7%, primarily involving pathogens such as *Mucor*, *Aspergillus*, and *Candida* species [16].

Timely and accurate identification of these fungal pathogens is essential for effective treatment; however, diagnostic challenges hinder early detection. Delayed treatment, particularly for invasive candidiasis, can increase mortality rates by 20% [178, 179]. Existing diagnostic methods, including microscopy, culture, antigen or antibody testing, and molecular assays, are limited by factors such as laboratory personnel expertise and the potential for false-positive results [79–81].

Advancements in molecular techniques and antigen detection offer alternatives to traditional diagnostics. Methods like PCR and mass spectrometry show promise for rapid and accurate identification of fungal infections, yet issues such as contamination and cost-effectiveness remain significant challenges. In resource-limited settings, environmental factors further complicate the management of IFIs, underscoring the need for improved diagnostic approaches.

In humid environments, such as India, conditions favor the growth of *Mucor* spp., especially in overcrowded settings with limited healthcare resources. The emergence of species like *Candida auris*, which exhibit high resistance to various disinfectants, presents additional challenges, particularly during the COVID-19 pandemic and in the context of chronic infectious diseases.

Looking ahead, addressing the challenges and limitations associated with future viral infections will be paramount. Key obstacles include the following:

1. Resource constraints: Many healthcare systems, particularly in LMICs, face significant limitations in resources, infrastructure, and trained personnel, which can hinder timely diagnosis and treatment of viral infections.
2. Diagnostic limitations: Current diagnostic methods may not be sufficiently rapid or sensitive to detect emerging viral pathogens early, leading to delays in intervention and increased transmission rates.
3. Viral variability: The ability of viruses to mutate rapidly poses a challenge for vaccine development and effectiveness, as well as for diagnostic accuracy.
4. Public health infrastructure: Insufficient public health infrastructure can impede effective surveillance and response to outbreaks, making it difficult to implement timely interventions.
5. Antiviral resistance: Just as with fungal pathogens, the emergence of resistant viral strains can complicate treatment options and necessitate ongoing research and development of new antiviral agents.

Acknowledging these limitations and challenges is crucial for developing effective strategies to combat future viral infections. Further research and development are necessary to enhance the accuracy and accessibility of diagnostics, ultimately improving patient outcomes in the face of these growing threats.

## Data Availability

Data sharing is not applicable to this article as no new data were created or analyzed in this study.
